# Retrospective analysis of the clinical presentation and imaging of eight primary benign mediastinal schwannomas

**DOI:** 10.1186/s13104-021-05694-6

**Published:** 2021-07-21

**Authors:** Ramiro Sandoval-Macias, Irving Daniel Ortiz-Sanchez, Ana Lilia Remirez-Castellanos, Luis Mora-Hernandez, Candelaria Cordova-Uscanga, Alejandra Mantilla-Morales, Tania Alejandra Galindo-Garcia, Armando Gamboa-Dominguez, Fernando Candanedo-Gonzalez

**Affiliations:** 1 AFINES Program to Support and Promote Student Research, Medical School, Ciudad Universitaria, UNAM, Mexico City, Mexico; 2 Department of Radiology, UMAE Hospital de Oncologia, Centro Medico Nacional Siglo XXI, IMSS, Mexico City, Mexico; 3 Department of Pathology, UMAE Hospital de Oncologia, Centro Medico Nacional Siglo XXI, IMSS, Av. Cuauhtemoc No. 330, Col. Doctores, Delegacion Cuauhtemoc, CP 06725 Mexico City, Mexico; 4 Department of Pathology, Instituto Nacional de Ciencias Medicas y Nutricion Salvador Zubiran, Mexico City, Mexico

**Keywords:** Schwannoma, Mediastinum, Computed tomography, Histological analysis, Immunohistochemistry, Neurogenic markers, Treatment, Follow-up

## Abstract

**Objective:**

Mediastinal schwannomas are sometimes confused with other neoplasms during initial radiological studies, especially when there is a history of cancer in another area. In these cases, a more accurate analysis using computed tomography (CT) or even magnetic resonance (MRI) is required. Our study aimed to perform a retrospective analysis of the clinical and imaging features for a series of patients with mediastinal schwannomas that were confirmed by histology and immunohistochemistry.

**Results:**

We found eight patients, five men and three women, with an average age of 51 years for this study. The main signs and symptoms at diagnosis were chest pain, dyspnea, cough, and dysphagia. CT showed that the tumor was located in the posterior compartment of the chest in 7/8 cases. Tumors > 10 cm were more heterogeneous and showed cystic changes. All patients underwent posterolateral thoracotomy, and radiological follow-up showed no evidence of recurrence. Histological analysis was considered the gold standard to confirm diagnosis, along with at least one neurogenic IHC marker. In conclusion, mediastinal schwannomas are benign encapsulated tumors. According to CT, schwannomas > 10 cm show cystic degeneration more frequently. Posterolateral thoracotomy allows complete resection and is considered the surgical approach of choice.

**Supplementary Information:**

The online version contains supplementary material available at 10.1186/s13104-021-05694-6.

## Introduction

Schwannomas are benign neoplasms that arise from Schwann cells, which surround peripheral nerve fibers [1, 2]. Less than 9% of schwannomas occur in the mediastinum, and only 5% of mediastinal neoplasms correspond to schwannomas [3, 4]. They often originate at the root of a spinal nerve and may involve the thoracic nerve. More than 90% of these lesions are unique and sporadic [3], and their highest incidence occurs between the fourth and sixth decades of life [3]. Schwannomas are characterized by slow growth and lack of specific symptoms. Therefore, a common presentation occurs as a well-defined lesion without a mass effect while performing radiographic studies focused on addressing various other complaints. However, when schwannomas are giant, it is possible for them to present as localized pain [5–8]. There are few studies in the Mexican population that analyze the characteristics of mediastinal schwannomas [9]. Our goal was to perform a retrospective analysis of the clinical and tomographic features of patients with primary benign mediastinal schwannoma in Mexico.

## Main text

### Material and methods

#### Study design and population characteristics

A retrospective and descriptive study was performed for patients with a mediastinal schwannoma diagnosis at UMAE Hospital de Oncologia, from January 2013 to January 2015. Permission was requested to access the clinical file to review patient records. Age, gender, time of evolution, type of treatment, and follow-up data were obtained from medical records. In all cases, telephone calls were made to complement follow-ups. Association with neurofibromatosis type 2, schwannomatosis, and Gorlin–Koutlas syndrome was also investigated. Chest CT and MRI scans from each patient were obtained from the radiology files and re-revaluated by two experienced radiologists (ALRC and LMH) who investigated the location, shape, size, and features of each lesion. In addition, a comparative analysis was made between the preoperative imaging studies (CT and MRI) and those performed during the follow-up of each patient after surgery to rule out disease recurrence. All cases were reevaluated by three experienced pathologists (CCU, AMM and FCG) using the WHO criteria [10].

#### Immunohistochemistry

All tissues were fixed in formaldehyde buffered at 10% and paraffin embedded. Histological sections were stained with hematoxylin and eosin. For immunohistochemistry (IHC) analysis, 5-μm sections of a representative block were obtained for each case. The following antibodies were used: S100 protein (Biosb), glial fibrillar acid protein (GFAP; Invitrogen), CD34 (Biocare), and Ki-67 (Biosb), which were used for detection on an automated immunostainer (Ventana, Biotek System Tucson, Ariz), and ran with appropriate simultaneous positive and negative controls.

### Results

#### Clinical features

During the period analyzed, 163 primary soft tissue tumors of the mediastinum were resected, of which only eight corresponded to mediastinal schwannomas (5%). Five men (63%) and three women (37%) with average age of 51 years (range 32–78 years). The average age for women was 43 years versus 56 years for men. Seventy-five percent of the patients had symptoms. The clinical manifestations were chest pain in four patients (64%), dyspnea (17%), cough (17%) and dysphagia (17%), in one patient respectively. Two patients were asymptomatic (25%). No weight loss was found for any of the patients. The average clinical evolution time was 4 months (ranging from 1 to 6 months). No associations with neurofibromatosis type 2, schwannomatosis, or Gorlin–Koutlas syndrome were found for any of these cases (Additional file 1: Table S1; Additional file 2: Table S2).

#### Imaging features

All patients had a chest X-ray and a chest CT, in which the lesion was identified. Chest X-ray films showed a well-defined round radiopaque mass in all cases. Only in three cases, a diagnosis of schwannoma was suspected at the time of CT. In one patient, the mediastinal tumor was identified as an asymptomatic finding secondary to a kidney transplant check-up. Another one of the patients had a history of clinical stage IIB breast cancer, in which follow-up with chest X-ray revealed a mediastinal mass in the left hemithorax. Initially this mass was thought to be due to disease recurrence. However, this was ruled out by performing a CT scan in which the schwannoma diagnosis was proposed, thus leading to a surgical approach for the patient.

**Fig. 1:**
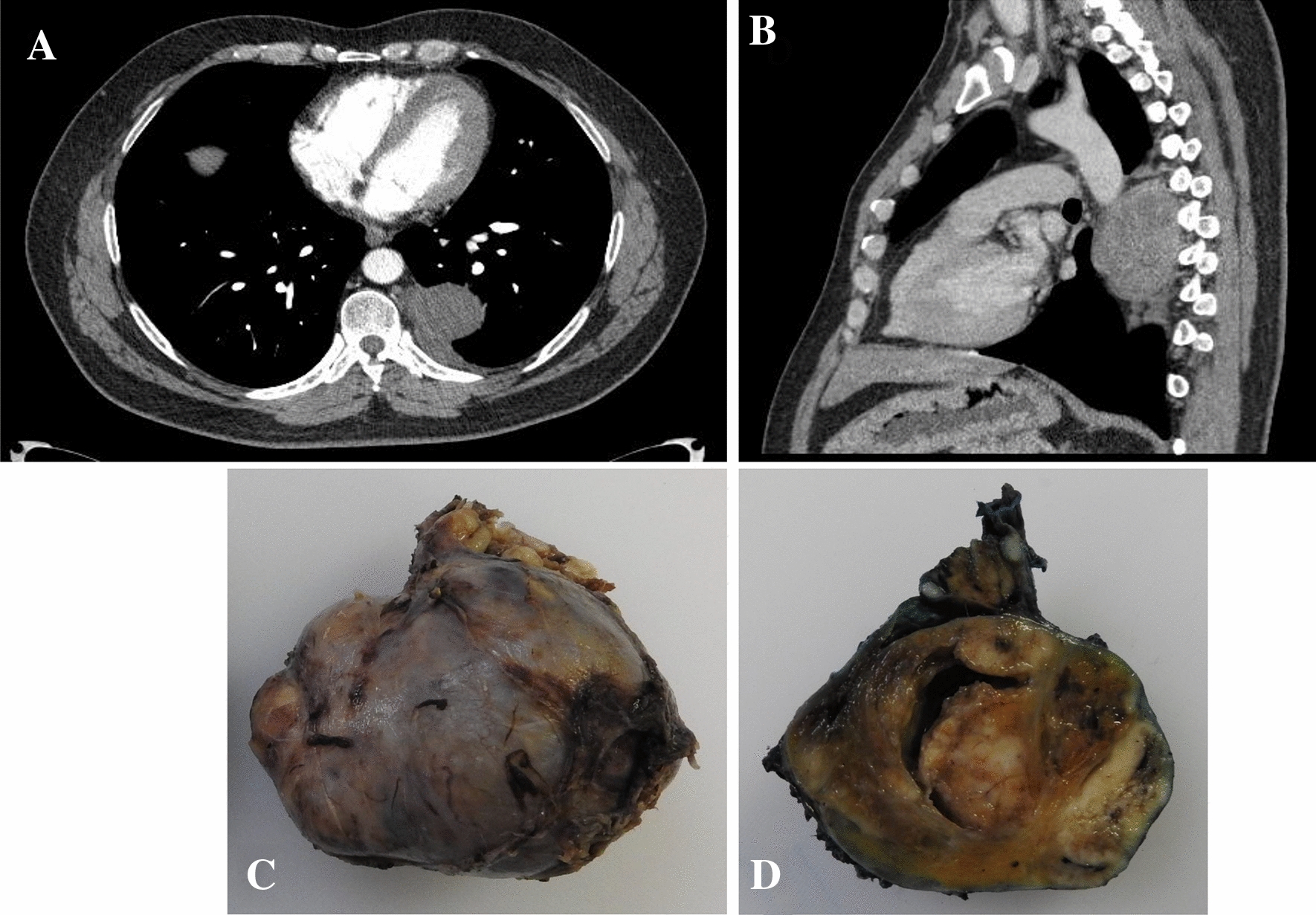
Benign mediastinal schwannoma. **a**, **b** CT images. The tumor was located in the posterior mediastinum and showed heterogeneous reinforcement with hypo and hyperintense areas after the application of contrast material; **c** Macroscopic images of the well-encapsulated ovoid tumor with a smooth surface, gray-yellow color; **d** Cross section of the tumor shows solid areas with cystic degeneration

For seven patients, the tumor was located in the posterior mediastinum (88%) according to CT, and in one case the mass was located in the posterior upper para-esophageal compartment. No patient showed evidence of tumoral activity elsewhere. In all cases, the mass was ovoid and well-defined. In four cases, the lesion was homogeneous, and in the other four cases it showed heterogeneous reinforcement with hypo and hyperintense areas after the application of contrast material (Fig. 1a, b). During follow-up, CT or MRI scans were performed in each patient, and no evidence of recurrent tumor activity was found. Only postsurgical changes were identified (Additional file 3: Fig. S1).

#### Pathologic findings

Macroscopically, all cases were solitary tumors, with ovoid growth, encapsulated and with a gray-yellow outer surface. The average size of the tumors was 8 cm (range 3.5–14.0 cm). The average size of the tumors for the group of women was 8.6 cm (range 4.0–14.0 cm) versus 7.16 cm in the case of men (range 3.5–11.0 cm). Fifty percent of the tumors showed cystic degeneration. These tumors had an average size of 10.0 cm (ranged between 7.0 and 14.0 cm), in contrast to tumors without cystic changes, which had an average size of 5.4 cm (ranges between 3.5 and 8.0 cm) (Fig. 1c, d). All cases were completely resected, and none presented necrosis.

#### Histopathological features

**Fig. 2:**
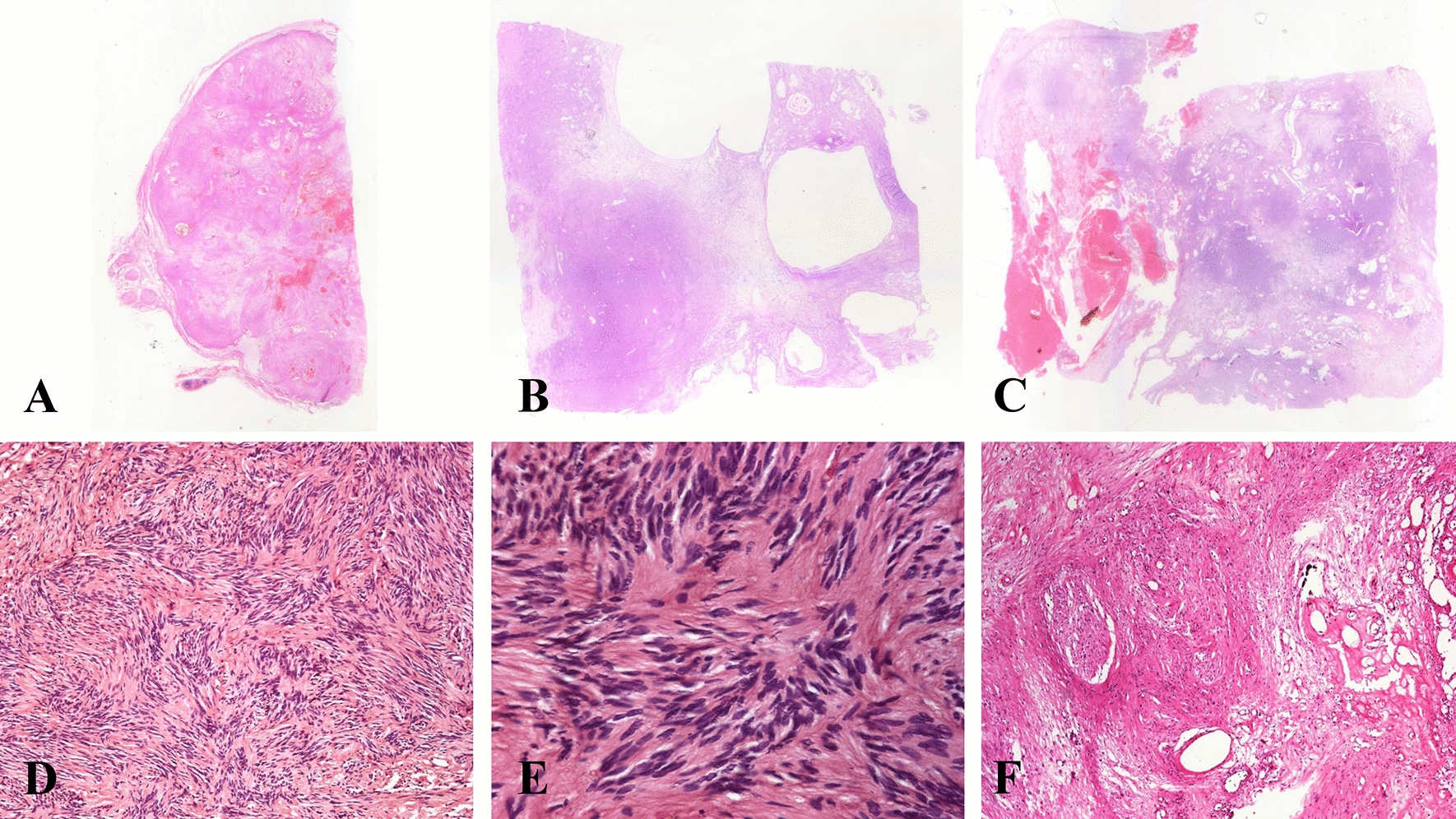
Microscopic images of conventional mediastinal schwannoma. **a** Encapsulated tumor with presence of nerves on the external surface; **b** Solid areas with cystic degeneration; **c** Areas of hemorrhage and vascular congestion; **d** Antoni A and Antoni B areas with biphasic patterns; **e** Verocay body, formed palisaded Schwann cells; **f** Hyalinized thick-walled vessels

Histologically, a small nerve fragment was identified in the extracapsular portion for only three of the cases (38%), (Fig. 2a). Four cases presented cystic changes (50%; Fig. 2b), six cases presented myxoid areas (75%), and in five cases there was hemorrhage (Fig. 2c). All cases showed hypercellular areas forming short bundles (Antoni A) alternating with scarce palisade formation (Verocay bodies) with a hypocellular component (Antoni B) (Fig. 2d). The neoplastic cells were spindle-shaped, with twisted nuclei and indistinct cytoplasmic borders, immersed in a collagenized stroma (Fig. 2e). One case (case #2) showed senescent changes with greater nuclear atypia and hyalinized stroma (Fig. 2f).

#### Immunohistochemistry findings

**Fig. 3:**
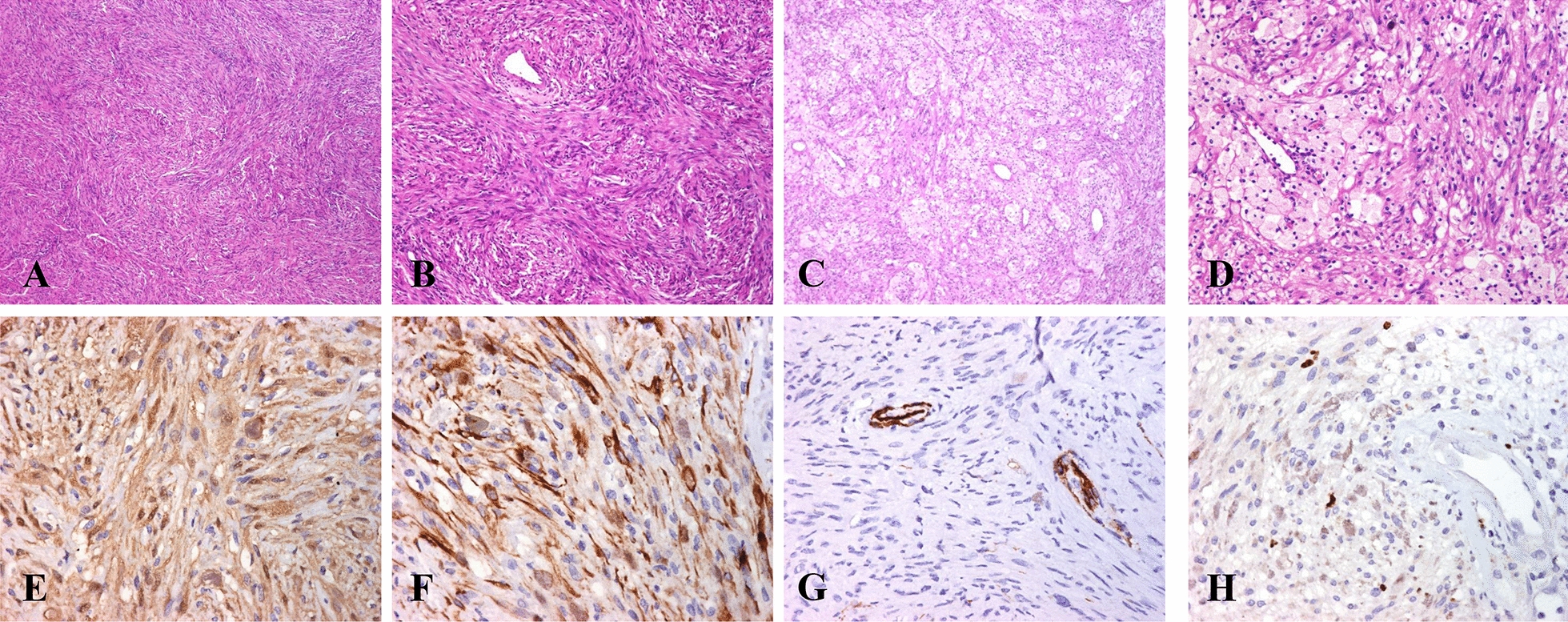
Microscopic images of cellular mediastinal schwannoma. **a** Tumor shows hypercellular areas; **b** Cellular schwannoma with storiform growth pattern mimicking solitary fibrous tumor; **c**, **d** Groups of numerous histiocytes; **e** Diffuse expression of S100 protein in neoplastic cells using IHC; **f** Intense expression of GFAP in neoplastic cells using IHC; **g** Neoplastic cells were negative for CD34; **h** The neoplastic cells show a proliferation index of 1% (Ki-67)

By IHC, all cases were intensely positive for the S100 protein in nucleus and cytoplasm, and showed a focal expression of GFAP, mainly in Antoni A areas. None of the cases showed CD34 expression. The proliferation index according to Ki-67 was less than 1% in all cases. One case (case #5) showed hypercellularity with a storiform pattern in approximately 50% of the tumor, mimicking a solitary fibrous tumor. However, the neoplastic cells were positive for two neurogenic markers (S100 protein and GFAP) and negative for CD34 (Fig. 3).

#### Treatment and follow-up of patients

All the patients underwent surgery for posterolateral thoracotomy and were followed up with radiology. In four cases, the thoracotomy was on the left-side versus three right-sided cases. For all patients, the tumor was resected completely. Only one patient was not available for follow-up. The mean follow-up of the patients was 67 months (ranges between 0 and 97 months) post-surgery. Four patients were found alive without clinical or radiological recurrence of the disease at their last visit. One patient died from diabetes mellitus (DM) complications, with no mediastinal tumor activity during the 72-month follow-up. There was no malignant transformation of the disease diagnosed for any of the patients.

### Discussion

Less than 9% of all schwannomas occur in the mediastinum [3, 4, 11]. In their epidemiological analysis of 1005 primary mediastinal neoplasm cases, Szolkowska et al. [4], found that only 5% of mediastinal neoplasms correspond to schwannomas. In agreement with these findings, schwannomas represented 5% of all soft tissue tumors of the mediastinum in our series. More than 90% of schwannomas are solitary, and sporadic neoplasms. Affect both genders equally, and may be occur in all age groups, with a higher incidence between the fourth and sixth decades of life [1–3]. In our series, the average age was 51 years, and the mean age for women was roughly a decade younger than for men.

Mediastinal schwannomas can originate from spinal, paravertebral sympathetic branches of vagus, phrenic and intercostal nerves, respectively [1–3, 12]. In our study, half of the cases originated in the paravertebral region. The classic presentation was an incidental asymptomatic mass found on routine investigations, such as a simple chest X-ray [13]. In our study, and in contrast to other international publications, 57% of the patients had symptoms attributable to a mediastinal schwannoma.

CT and MRI can help to accurately determine the exact location of the mediastinal tumor and its relationship to adjacent structures. On CT scanning, schwannomas appear a as well-defined rounded mass with smooth margins, low densities and mild enhancement in the paravertebral region, and long along the courses of intercostal nerves [5–8, 14]. They have a variable enhancement pattern after administration of intravenous contrast, including multiple hypodense or cystic areas with central hypodensity and central enhancement, as well as peripheral hypodensity [14, 15]. Calcifications are occasionally detected, and low attenuation correlates with areas of hypocellularity, cystic change, hemorrhage, and lipidization [3, 16].

Typically, mediastinal schwannomas measure less than 10 cm. However, there are a few case reports of giant mediastinal schwannomas that have measured between 13 and 23 cm [5–8]. These cases are more frequently associated with the presence of symptoms such as cough and chest pain. Giant schwannomas are more heterogeneous and show cystic degeneration [7]. In agreement with Quartey et al. [7] in our series cystic degeneration was observed more frequently in tumors larger than 10 cm. Tumors smaller than 10 cm were more frequently homogeneous with solid areas in the absence of cystic degeneration. This supports the idea that degeneration changes are directly related to the size of the tumor.

Mediastinal schwannomas can be misdiagnosed as lymph node metastasis [17]. The clinical distinction between mediastinal schwannoma and metastasis in cases where there is a history of cancer can be difficult, and requires surgical removal followed by histopathological analysis. The location and composition obtained by means of imaging studies are critical to make a differential diagnosis. Even when using sophisticated studies, such a ^18^F-fluorodeoxyglucose positron emission tomography and computed tomography (^18^F-FDG PET/CT), mediastinal schwannomas can simulate metastases of breast cancer lymph nodes, as shown by Martinez-Esteve et al. [17]. Similarly, we found a patient with a clinical history of stage IIB breast cancer during follow-up, which showed a mediastinal mass on a chest X-ray that was initially thought to be a recurrence of the disease. However, when a CT scan was performed, the origin site for the tumor was defined precisely and proposed as schwannoma. After resection, the histopathological analysis showed a schwannoma with diffuse expression of S100 protein and GFAP.

In 1981, Woodruff et al. [18] described 14 cellular schwannomas, including a variant with compact cellular growth predominantly in Antoni A areas composed of interlaced fascicles and variable mitotic activity, in absence of Verocay bodies, hyperchromasia, and pleomorphism. This variant represented 4.6% of all benign peripheral nerve sheath tumors, usually with a paravertebral, pelvic, retroperitoneal, or mediastinal location [19], and can be confused with a malignant peripheral nerve sheath tumor or with a solitary fibrous tumor [20, 21]. In our study, we found only one case of a cellular schwannoma with a storiform pattern mimicking a solitary fibrous tumor. However, the schwannoma diagnosis was supported by light microscopy and corroborated by S100 protein and GFAP expression and CD34 negative.

Schwannomas are slow-growing tumors with a proliferation rate of 2% to 3% [19, 22]. However, cellular schwannomas usually present ≥ 5 mitoses in 10 high power fields (HPF), and therefore, can be confused with malignant tumors. Accordingly, Abe et al. [22] observed a higher proliferation rate between cellular versus ordinary schwannomas (8.2% vs 5.2%, respectively), and Casadei et al. [22] studied the proliferation index for cellular schwannomas and found no usefulness of this parameter as a prediction maker for disease recurrence. In contrast, all schwannomas in our study showed a proliferation index of ≤ 1% and none of them recurred or metastasized.

## Conclusion

Mediastinal schwannomas are benign encapsulated tumors, and are more frequently located in the posterior compartment when using CT. Tumors > 10 cm are usually heterogeneous with cystic degeneration. Histological analysis remains the gold standard to confirm the diagnosis along with IHC markers that include at least one neurogenic marker.

## Limitations

This is a retrospective study with a small sample.

## Supplementary Information

**Additional file 1: Table S1.** Summarizes the clinical and image characteristics of all patients.

**Additional file 2: Table S2.** Morphological characteristics of eight cases of benign schwannomas of the mediastinum.

**Additional file 3: Fig S1.** Mediastinal schwannoma (Case 7): a) Preoperative MRI for T2 sequence shows a right paravertebral mediastinal mass, which was ovoid with smooth edges, solid, and measuring 89 × 70 × 55 mm; b) Heterogeneous reinforcement after the application of intravenous contrast, without pleural infiltration, to either vertebral bodies or vascular structures; c) Follow-up CT three years after surgery only shows focal pleural thickening, with a homogeneous oval appearance in the upper right lobe posterior segment, measuring 3.4 × 1.6 cm, and a mean density of 10 HU (red asterisk), compatible with scar, and without evidence of tumor activity.

## Data Availability

The datasets used and/or analysed during the current study available from the corresponding author on reasonable request.

## Abbreviations

AFINES: Program to support and promote student research
CT: Computed tomography
^18^F-FDG PET/CT: ^18^F-fluorodeoxyglucose positron emission tomography and computed tomography
GFAP: Glial fibrillary acid protein
IHC: Immunohistochemistry
MRI: Magnetic resonance imaging
UMAE: Unidad Medica de Alta Especialidad
UNAM: Universidad Nacional Autonoma de Mexico
X-ray: Radiograph
