# Supplementary material for: Retrospective analysis of the clinical presentation and imaging of eight primary benign mediastinal schwannomas
Source: BMC Res Notes. 2021 Jul 21;14:278. doi: 10.1186/s13104-021-05694-6 (PMC8296632; doi:10.1186/s13104-021-05694-6)
Supplement: Supplementary file 1 — Additional file 1: Table S1. Summarizes the clinical and image characteristics of all patients. [file 13104_2021_5694_MOESM1_ESM.docx]

**Table S1. Summarizes the clinical and image characteristics of all patients.**

**Case # Age Background Location of the tumor Image features Macroscopic appearance**

1 59 Breast cancer. Third and fourth left CT. Tumor of 8.0 cm,

Hemitorax pain. intercostal space. Homogeneous. solid with areas of

hemorrhage.

2 53 Anal fistula. Left paravertebral in T4-T5. CT. Tumor of 6.0 cm,

Hemitorax pain. Homogeneous. solid with areas of

hemorrhage.

3 57 Kidney transplant Paravertebral in T8. Heterogeneous, hyperintense Tumor of 3.5 cm,

protocol. in T2 with homogenous solid with areas of

Asymptomatic. reinforcement. hemorrhage.

4 49 Dyspnea. Paravertebral tumor between CT. Predominantly hypodense, Tumor of 11.0 cm,

5th and 8th posterior left costal heterogeneous with higher solid with cystic and

space. density areas. In the simple myxoid changes.

phase, the relative density

of mass was 26.6 HU.

The hypodense

areas of 40.14 HU.

5 31 Hemitorax pain. CT. Cystic and myxoid changes Heterogeneous, hyperintense.

6 78 Hemitorax pain. Right dorsal column dependent Heterogeneous, hyperintense. Solid with cystic changes.

on emerging nerve root at level

T8 to T11.

7 41 Paraesophageal in CT. Heterogeneous, Solid with cystic changes.

postero-superior mediastinum. predominantly isodense

Right paravertebral. in arterial and venous

phases with an attenuation

value of 20-36 HU.

8 38 Asymptomatic. CT. Homogeneous. Solid with areas of

hemorrhage.

CT: computed tomography; HU: Hounsfield units
