# Supplementary material for: Retrospective analysis of the clinical presentation and imaging of eight primary benign mediastinal schwannomas
Source: BMC Res Notes. 2021 Jul 21;14:278. doi: 10.1186/s13104-021-05694-6 (PMC8296632; doi:10.1186/s13104-021-05694-6)
Supplement: Supplementary file 2 — Additional file 2: Table S2. Morphological characteristics of eight cases of benign schwannomas of the mediastinum. [file 13104_2021_5694_MOESM2_ESM.docx]

**Table S2. Morphological characteristics of eight cases of benign schwannomas of the mediastinum.**

**Size tumor**

**Morphological features < 10 cm >10 cm**

**N=6 (%) N=2 (%)**

Cystic changes 2/6 (33%) 2/2 (100%)

Antoni-A areas 6/6 (100%) 2/2 (100%)

Antoni-B areas 6/6 (100%) 2/2 (100%)

Verocay bodies 6/6 (100%) 1/2 (50%)

Hypercellular areas 0/6 (0%) 1/2 (50%)

Myxoid stroma 4/6 (67%) 2/2 (100%)

Blood vessels hailinized 5/6 (83%) 2/2 (100%)

Hyalinized stroma 2/6 (33%) 1/2 (50%)

Senescent changes 1/6 (17%) 0/2 (0%)

Lymphoid nodules 5/6 (83%) 2/2 (100%)

Presence of histiocytes 1/6 (17%) 1/2 (50%)

Presence of hemosiderin 6/6 (100%) 2/2 (100%)

Identification of nerve in the periphery 3/6 (50%) 0/2 (0%)
