# Supplementary figures and images for: Retrospective analysis of the clinical presentation and imaging of eight primary benign mediastinal schwannomas
Source: BMC Res Notes. 2021 Jul 21;14:278. doi: 10.1186/s13104-021-05694-6 (PMC8296632; doi:10.1186/s13104-021-05694-6)

## Slide 1
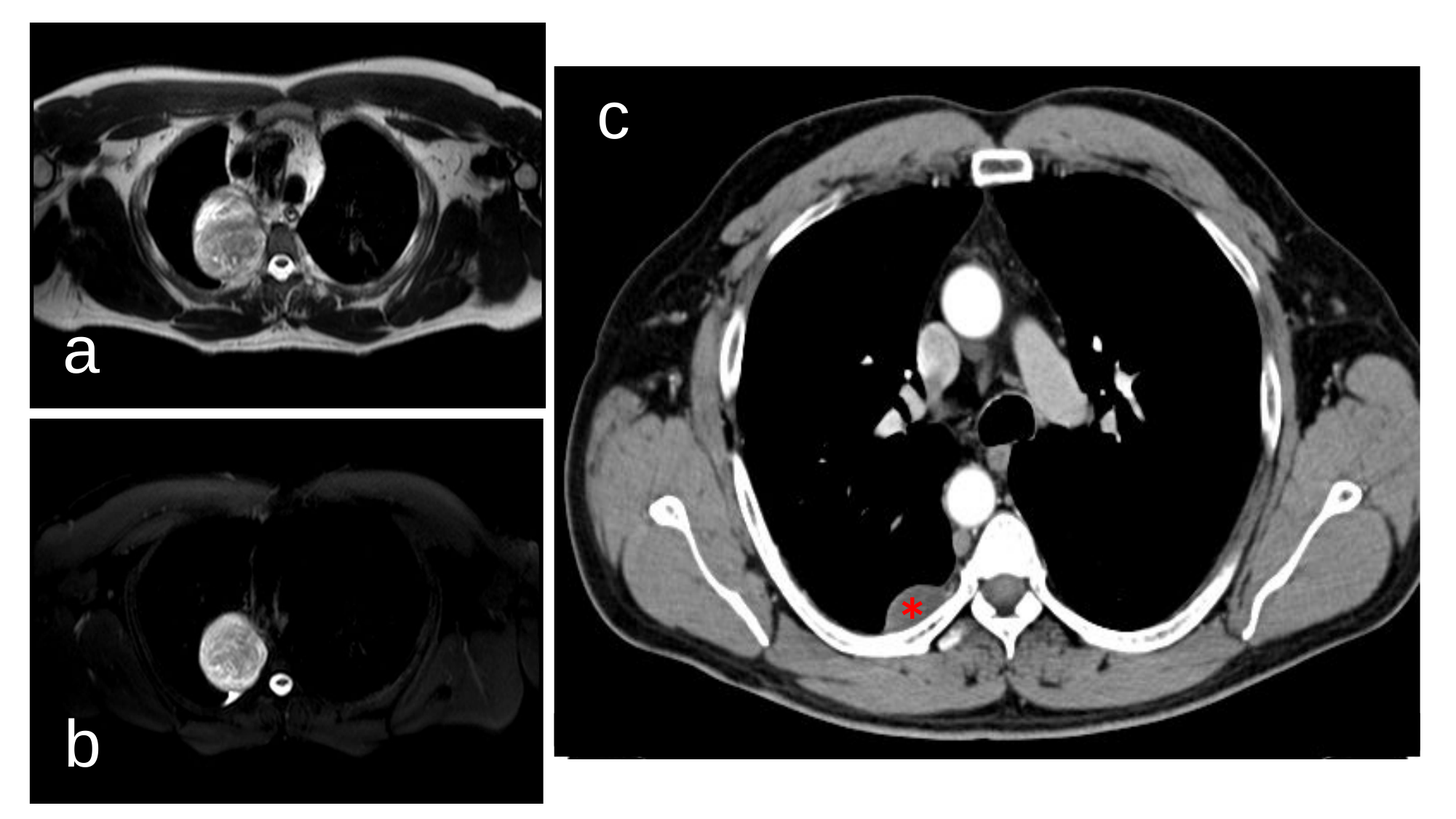

a
b
c
*

Supplement: Supplementary file 3 — Additional file 3: Fig S1. Mediastinal schwannoma (Case 7): a) Preoperative MRI for T2 sequence shows a right paravertebral mediastinal mass, which was ovoid with smooth edges, solid, and measuring 89 × 70 × 55 mm; b) Heterogeneous reinforcement after the application of intravenous contrast, without pleural infiltration, to either vertebral bodies or vascular structures; c) Follow-up CT three years after surgery only shows focal pleural thickening, with a homogeneous oval appearance in the upper right lobe posterior segment, measuring 3.4 × 1.6 cm, and a mean density of 10 HU (red asterisk), compatible with scar, and without evidence of tumor activity. [file 13104_2021_5694_MOESM3_ESM.pptx]
